# Severe Symptomatic Hypercalcemia in a Patient With Familial Hypocalciuric Hypercalcemia

**DOI:** 10.7759/cureus.20057

**Published:** 2021-11-30

**Authors:** Roshini Kurian, Gagan Madegowda Chandrashekar, Mc Anto Antony, Lakshya Chandra, Ravi Kant

**Affiliations:** 1 Chemical Pathology, University Hospitals of Leicester, National Health Service Trust, Leicester, GBR; 2 Gastroenterology, University Hospital of North Durham, Durham, GBR; 3 Endocrinology, Diabetes and Metabolism, Medical University of South Carolina, Anderson, USA; 4 Internal Medicine, Bon Secour St. Francis Hospital, Greenville, USA

**Keywords:** casr mutation, urine calcium, zolendronic acid, cinacalcet, corrected serum calcium, familial hypocalciuric hypercalcemia

## Abstract

One of the less common causes of hypercalcemia is familial hypocalciuric hypercalcemia (FHH). It is an autosomal-dominant genetic condition, which presents asymptomatically in most patients while some may have mild symptoms. The serum calcium levels are mildly elevated with mild elevation in parathyroid hormone, which rarely requires management with pharmacologic agents. We present an unusual case report of a 76-year-old woman, confirmed to have FHH type 1 mutation, presented with symptomatic hypercalcemia probably set off by metabolic stresses of her age and needing intensive treatment with intravenous bisphosphonates, calcitonin and cinacalcet.

## Introduction

Familial hypocalciuric hypercalcemia (FHH) type 1 is a rare disorder inherited as an autosomal-dominant trait, characterized by an inactivating mutation in the calcium-sensing receptor (CASR) gene. This syndrome most often presents clinically as a mild, persistent, and asymptomatic elevation in serum calcium levels with normal to mildly elevated serum parathyroid hormone. FHH types 2 and 3 arise from mutations in other genes involved in calcium homeostasis and present similarly [1,2].

We present a case of a 76-year-old Caucasian female with FHH type 1 and a documented CASR mutation who presented with severe symptomatic hypercalcemia, an unusual presentation of this condition.

## Case presentation

Our patient is a 76-year-old Caucasian female with a past medical history of unexplained hypercalcemia and mild osteopenia. She was initially seen in our clinic for osteoporosis, during which time she disclosed to us that she believed hypercalcemia ran in her family. Of note, she also disclosed that she had not seen a physician until she was 60 years old. Labs obtained in our clinic showed an elevated corrected (for albumin) serum calcium of 12.2 mg/dL (8.6-10.0 mg/dL). Repeat lab work obtained one month later demonstrated a corrected serum calcium level of 11.6 mg/dL in the setting of elevated parathyroid hormone (PTH) 136 pg/mL (15-65 pg/mL), low serum phosphorus 2.3 mg/dL (2.5-4.9 mg/dL) and an undetectable 24-hour urinary calcium <17 mg/24 h (100-300 mg/24 h). A nuclear medicine sestamibi single-photon emission computed tomography (SPECT) parathyroid scan demonstrated no evidence of parathyroid adenoma. Given high suspicion of FHH, CASR gene sequencing was performed that revealed a heterozygous sequence change with a c.2039G>A mutation in exon 7 of the CASR, confirming the diagnosis of FHH type 1. Unfortunately, after the diagnosis, patient was lost to follow up.

Two years later, the patient presented to the emergency department with altered mental status. Initial laboratory evaluation revealed the presence of a urinary tract infection (UTI) with a significantly elevated corrected serum calcium level 13.2 mg/dL and an elevated serum PTH 113.7 pg/mL. Treatment began with an intravenous (IV) antibiotic along with a combination of IV fluids and IV furosemide. This treatment regimen was maintained over the course of three days, over which time the patient continued to experience altered mental status despite bloodwork indicating that she was clearing the infection (Tables 1, 2). Her corrected serum calcium levels continued to be elevated, sometimes as high as 14.1 mg/dL, despite fluid resuscitation and diuretic therapy. Due to lack of improvement in clinical status, the patient underwent a comprehensive evaluation which came back unremarkable as summarized in Table 3. The initial as well as the repeat MRI brain revealed moderate cerebral atrophy with no acute changes. It became evident that her symptoms were most likely due to hypercalcemia. Patient continued to receive IV fluids and IV furosemide, with no improvement in her clinical status and serum calcium levels, with corrected serum calcium levels ranging consistently above 11.4 mg/dL and reaching a value of 14.1 mg/dL on day 9 of hospitalization. At this time, endocrinology was consulted and the decision was then made to initiate intensive calcium-lowering therapy.

**Table 1:** Intial Laboratory Work-Up

Urine	On Presentation (In-Patient)
Nitrates	Positive
Leukocyte Esterase	Large
Urine Bacteria	Moderate
Urine Culture	No Growth

**Table 2:** Comparison of Laboratory Work-Up on Presentation and on Day 3 of Treatment Initiation

Blood Counts	On Presentation (In-Patient)	Day 3 of IV Antibiotic (In-Patient)
Total White Cell Count (4-11 x 10^9^/L)	15.63	10.64
Absolute Neutrophil Count (1.5-7.5 x 10^9^/L)	9.15	6.50
Neutrophils (%)	58.6	61.6

**Table 3:** Additional Work-Up for Hypercalcemia and Altered Mental Status SPEP - serum protein electrophoresis; TSH - thyroid-stimulating hormone; ANA - antinuclear antibody; CSF - cerebrospinal fluid; VDRL- venereal disease research laboratory test

Investigation	Result
SPEP for Myeloma	No Monoclonal Band Identified
TSH (0.300-4.00 uIU/mL)	1.050
Rheumatoid Factor (0-14 IU/mL)	<10
ANA	Negative
CSF Microscopy, Culture	No Growth
CSF VDRL	Non-reactive

Cinacalcet 30 mg PO twice daily and IV calcitonin were started while continuing rehydration and diuretic therapy. Two days later, the patient’s altered mental status and hypercalcemia were still unimproved. The corrected serum calcium was 12.7 mg/dL on day 12 and a one-time dose of IV zoledronic acid was administered along with increased calcitonin dose. The patient’s mental status began to show signs of improvement with this regimen. Both a parathyroid ultrasound and repeat nuclear medicine sestamibi SPECT parathyroid scan on day 13 demonstrated no evidence of a parathyroid adenoma. Day 14 saw the patient’s corrected serum calcium level within reference range at 9.8 mg/dL, for the first time since her admission (Table 4). Over the next three days on this regimen, her mental status returned to baseline as her serum calcium levels normalized. Calcitonin was then discontinued and dose of cinacalcet was reduced, and the patient was discharged home on 30 mg cinacalcet PO once daily. No adverse effects from the cinacalcet were reported.

**Table 4:** Laboratory Trend During Treatment Course PTH - parathyroid hormone; ALP - alkaline phosphatase; eGFR - estimated glomerular filtration rate; NC - not checked

Parameters	Reference Range	First Presentation (Out-Patient)	Second Presentation (In-Patient)			
			Day 1	Day 9	Day 12	Day 14
Corrected Serum Calcium	8.6-10.0 mg/dL	11.6	13.2	14.1	12.7	9.8
PTH	15.0-65.0 pg/mL	136	113.7	NC	NC	NC
Phosphorus	2.5-4.5 mg/dL	2.3	NC	NC	NC	NC
ALP	35-104 U/L	NC	53	NC	60	NC
eGFR	>60 mL/min/1.73 m^2^	>60	>60	>60	>60	>60
Urine Calcium 24 h	100-300 mg/24 h	<17	NC	NC	NC	NC

## Discussion

FHH classically presents as a chronic, mild, and asymptomatic elevation in serum calcium with normal to moderately elevated serum PTH. However, this patient with a known CASR mutation presented with altered mental status in setting of UTI that only resolved when her severe hypercalcemia was corrected. Aggressive rehydration and diuretic therapy were not sufficient to lower the patient’s serum calcium levels. Cinacalcet 30 mg twice a day with bisphosphonate therapy and IV calcitonin were required to establish eucalcemia in this patient, and we observed improvement of her mental status as her serum calcium levels fell to within the reference range. 

There have been reports of hypercalcemia in children and adults with FHH treated successfully with cinacalcet [1,3]. Of the 12 cases reviewed [4-9], all had DNA sequence which confirmed FHH and were treated with cinacalcet. However, only seven out of 12 of them were symptomatic for the hypercalcemia, and symptoms included muscle cramps, poor wound healing, psychosis and poor memory. Achievement of eucalcemia with cinacalcet therapy at either 30 or 60 mg per day coincided with improvement of symptoms in these cases. In contrast, cinacalcet was not the only pharmacological agent used in our patient as her condition warranted aggressive management to achieve resolution of symptoms. Therefore, assessing the efficacy of cinacalcet alone in achieving normocalcemia was not possible. 

There are also case reports of the co-existence of FHH and primary hyperparathyroidism, which was reported in five patients [10-13]. All of these patients had a CASR gene mutation with a positive image finding suggestive of a parathyroid adenoma and underwent surgery which provided histological confirmation. Our patient underwent a parathyroid ultrasound scan and a nuclear medicine sestamibi SPECT parathyroid scan twice, which did not identify abnormal parathyroid mass. However, the sensitivity for a parathyroid ultrasound scan [14] is only about 74% and that of Tc-99m sestamibi SPECT scan [15] is between 80% and 91%. Hence, there is a possibility that we could have missed an adenoma. On the other hand, the 4DCT parathyroid scan [16] has a sensitivity between 58% and 92% depending on multi-gland or single-gland disease. The definitive confirmation would require a parathyroid explorative surgery and histopathological confirmation of any findings. Surgical consultation was provided to our patient. However, at the time of the consult, the patient had achieved good clinical improvement and therefore surgery was deferred. 

Our patient presented with an uncommon presentation of FHH with severe symptomatic hypercalcemia which required aggressive medical management. There has been a similar case report of symptomatic hypercalcemia in an elderly lady with FHH [17] where alendronic acid was used to alleviate symptoms. After achieving normocalcemia and clinical improvement back to baseline mental status, our patient was discharged on cinacalcet. However, she was able to use it only for a short duration due to financial constraints. Subsequently, she was offered alendronic acid which she refused fearing side effects. She was also offered to follow up with the surgeon to re-consider parathyroid explorative surgery which she declined.

Recommendations for treatment of symptomatic hypercalcemia in patients with FHH are limited by a lack of placebo-controlled trials demonstrating improvement of symptoms with cinacalcet therapy. There is also a potential problem of similar cases in which symptoms attributed to hypercalcemia did not improve and thus went unreported.

Patients with FHH have hypercalcemia their entire life. Therefore, it is interesting that our patient was not diagnosed with this condition until the age of 76 years. She was asymptomatic her entire life and may have become gradually symptomatic. It is possible that she may have had some baseline neurocognitive deficits from moderately elevated serum calcium but given the chronicity of hypercalcemia, these deficits may have been well compensated. We suggest that the acute UTI and the metabolic stresses of her age may have precipitated a gradual rise in the serum calcium concentration to the point where she became symptomatic.

## Conclusions

Our patient with FHH type 1 presented with altered mental status attributed to severe hypercalcemia. We believe our patient was hypercalcemic all her life and became symptomatic due to UTI and metabolic stressors related to her age. Her presentation, lack of improvement of mental status to IV fluids and diuretic warranted further treatment measures.

This unique case report highlights the diagnostic enigma posed by such a presentation and throws light on management with aggressive calcium-lowering therapies including cinacalcet, calcitonin and bisphosphonate.

## References

[1] Mayr B, Schnabel D, Dörr HG, Schöfl C (2016). Genetics in endocrinology: gain and loss of function mutations of the calcium-sensing receptor and associated proteins: current treatment concepts. Eur J Endocrinol.

[2] White E, McKenna J, Cavanaugh A, Breitwieser GE (2009). Pharmacochaperone-mediated rescue of calcium-sensing receptor loss-of-function mutants. Mol Endocrinol.

[3] Brown EM (2010). Clinical utility of calcimimetics targeting the extracellular calcium-sensing receptor (CaSR). Biochem Pharmacol.

[4] Rasmussen AQ, Jørgensen NR, Schwarz P (2011). Clinical and biochemical outcomes of cinacalcet treatment of familial hypocalciuric hypercalcemia: a case series. J Med Case Rep.

[5] Mogas E, Campos-Martorell A, Clemente M, Castaño L, Moreno-Galdó A, Yeste D, Carrascosa A (2018). Successful use of cinacalcet to treat parathyroid-related hypercalcemia in two pediatric patients. Endocrinol Diabetes Metab Case Rep.

[6] Timmers HJ, Karperien M, Hamdy NA, de Boer H, Hermus AR (2006). Normalization of serum calcium by cinacalcet in a patient with hypercalcaemia due to a de novo inactivating mutation of the calcium-sensing receptor. J Intern Med.

[7] Alon US, VandeVoorde RG (2010). Beneficial effect of cinacalcet in a child with familial hypocalciuric hypercalcemia. Pediatr Nephrol.

[8] Reh CM, Hendy GN, Cole DE, Jeandron DD (2011). Neonatal hyperparathyroidism with a heterozygous calcium-sensing receptor (CASR) R185Q mutation: clinical benefit from cinacalcet. J Clin Endocrinol Metab.

[9] Schwarz P, Larsen NE, Lønborg Friis IM, Lillquist K, Brown EM, Gammeltoft S (2000). Familial hypocalciuric hypercalcemia and neonatal severe hyperparathyroidism associated with mutations in the human Ca2+-sensing receptor gene in three Danish families. Scand J Clin Lab Invest.

[10] Eldeiry LS, Ruan DT, Brown EM, Gaglia JL, Garber JR (2012). Primary hyperparathyroidism and familial hypocalciuric hypercalcemia: relationships and clinical implications. Endocr Pract.

[11] Yabuta T, Miyauchi A, Inoue H, Yoshida H, Hirokawa M, Amino N (2009). A patient with primary hyperparathyroidism associated with familial hypocalciuric hypercalcemia induced by a novel germline CaSR gene mutation. Asian J Surg.

[12] Papadakis M, Meurer N, Margariti T, Meyer A, Weyerbrock N, Dotzenrath C (2016). A novel mutation of the calcium-sensing receptor gene in a German subject with familial hypocalciuric hypercalcemia and primary hyperparathyroidism. Hormones (Athens).

[13] Marstrand SD, Tofteng CL, Jarløv A, Borgwardt L, Schwarz P (2021). Concomitant familial hypocalciuric hypercalcemia and single parathyroid adenoma: a case report. J Med Case Rep.

[14] Tublin ME, Pryma DA, Yim JH, Ogilvie JB, Mountz JM, Bencherif B, Carty SE (2009). Localization of parathyroid adenomas by sonography and technetium tc 99m sestamibi single-photon emission computed tomography before minimally invasive parathyroidectomy: are both studies really needed?. J Ultrasound Med.

[15] Norton SK, Johnson LW, Griffen FD (2002). The sestamibi scan as a preoperative screening tool. Am Surg.

[16] Yeh R, Tay YD, Tabacco G (2019). Diagnostic performance of 4D CT and sestamibi SPECT/CT in localizing parathyroid adenomas in primary hyperparathyroidism. Radiology.

[17] Hashida H, Honda T, Morimoto H, Aibara Y, Matsumoto Y, Kurita M (2002). [An elderly case suspected of familial hypocalciuric hypercalcemia subsequent to manifestation of hypercalcemia]. Nihon Ronen Igakkai Zasshi.

